# An Android Inline Hooking Framework for the Securing Transmitted Data

**DOI:** 10.3390/s20154201

**Published:** 2020-07-28

**Authors:** Yu-an Tan, Shuo Feng, Xiaochun Cheng, Yuanzhang Li, Jun Zheng

**Affiliations:** 1 School of Computer Science and Technology, Beijing Institute of Technology, Beijing 100081, China; tan2008@bit.edu.cn (Y.-a.T.); 3220190793@bit.edu.cn (S.F.); popular@bit.edu.cn (Y.L.); 2 Institute of Artificial Intelligence and Blockchain, Guangzhou University, Guangzhou 510006, China; 3 Department of Computer Science, Middlesex University, London NW4 4BE, UK; Xiaochun.cheng@gmail.com

**Keywords:** sensitive information leakage, Android, native hook, ELF file format, VirtualXposed

## Abstract

Information leaks can occur through many Android applications, including unauthorized access to sensors data. Hooking is an important technique for protecting Android applications and add security features to them even without its source code. Various hooking frameworks are developed to intercept events and process their own specific events. The hooking tools for Java methods are varied, however, the native hook has few methods. Besides, the commonly used Android hook frameworks cannot meet the requirement of hooking the native methods in shared libraries on non-root devices. Even though some approaches are able to hook these methods, they have limitations or are complicated to implement. In the paper, a feasible hooking approach for Android native methods is proposed and implemented, which does not need any modifications to both the Android framework and app’s code. In this approach, the method’s reference address is modified and control flow is redirected. Beyond that, this study combines this approach with VirtualXposed which aims to run it without root privileges. This hooking framework can be used to enforce security policies and monitor sensitive methods in shared objects. The evaluation of the scheme demonstrates its capability to perform hook operation without a significant runtime performance overhead on real devices and it is compatible and functional for the native hook.

## 1. Introduction

Most Android devices have built-in sensors that measure motion, orientation, and various environmental conditions. As the most popular smartphone operating system worldwide [1], the Android platform supports three broad categories of sensors: motion sensors, environmental sensors, and position sensors [2,3]. Whatever the sensor is, Android allows the application to get the raw data from these sensors. Unfortunately, due to the open-source character and its popularity, there are a number of different threats to suffer from ongoing data leaking [4,5,6], such as malware [7,8], spyware, or the lack of fail-safe defaults in Android SDK [9]. Even benign apps can expose the users to various threats which contain vulnerable components [10], such as using insecure encryption, permission leaking [11,12,13], data are hijacked during transmission [14], and remote code execution [15]. Moreover, Android apps’ static analysis becomes more and more difficult nowadays because both benign and malicious developers use various protection techniques, such as code obfuscation [16] to prevent the apps from reversing. Different technologies for preventing information leaks have been proposed [17,18,19,20]. Hooking techniques can be used by developers to deploy new security extensions on their device or to analyze apps dynamically [21], which is able to avoid modification of the original code.

Currently, although there are various hooking tools for Java methods, there are few tools for Android native hook. One should notice that if we modify the App’s shared libraries directly to change the target method’s instructions, and repackage the application, we need to re-sign the App, which breaks the Android’s signature-based same origin policy and furthermore leads to legal concerns about illicit tampering with foreign code [22]. Regarding native hook, there are mainly two major routes, Procedure Linkage Table (PLT) Hook and Inline Hook. These hooking methods have their own advantages and disadvantages, and they are extremely different in principle and applicable scenarios. PLT hook directly modifies the Global Offset Table (GOT) in Executable and Linkable Format (ELF) files to jump to user-defined hook function code when calling external functions of the shared library [23]. It is less technically difficult, requiring only the calculation and modification of memory addresses. However, PLT and GOT tables contain only the shared library’s external functions that ELF needs to call. It has a drawback that the internal custom functions, so as not in the PLT table, cannot be hooked. The basic principle of Inline Hook is to insert jump instructions in a code segment to direct the program execution process to the functional code required by the user [24]. The advantage of this method is that it is completely independent of whether the function is in the PLT table. However, there may be different compatibility and stability issues for processors of different architectures, processor instruction sets, compiler optimization options, and operating system versions. In addition, it may be formidable to analyze and locate the problems. The inline replacement bytes for ARM are 8–14 bytes, much higher than the 5 bytes for x86 platforms. When the function is relatively short, it does not work.

Due to the limitations of the current native hooking methods, this paper will put forward a feasible and effective Android native hook method. This method can hook both external methods and inner user-defined functions, consequently, it has a wide range. The idea of this hooking method is tampering the reference address in the relocation section which saves the offsets of the methods defined in the .text section. The sections which have the methods’ reference contain .got which stores the entry address of external invocation, .data.rel.ro which has the vtable of C++ class and has the pointer to the virtual methods and other relocation section. This hooking method is achieved by absolute address calculation and rewriting this address to the new function’s address to redirect control flows to the patch code. This hooking method does not require register calculation and assembly code modification but only needs memory address calculation and modification, accordingly it has low technical difficulty. Since this method can hook C++ inner functions, using it can make up for the lack of the PLT method.

## 2. Background

Android applications are implemented in java, compiled into DEX bytecode. Android programs are executed in either the Dalvik virtual machine or the Android Runtime (ART). Developers use a series of tools of Native Development Kit (NDK) to quickly develop C/C++ dynamic library, and automatically package the dynamic link library and Java applications into Android Package (APK). Java Native Interface (JNI) is a native programming interface, a mechanism provided by the Java language for Java and C/C++ mutual calls. Shared object (SO) files are dynamic link files, and their format is the ELF file.

### 2.1. ELF File Structure

Similar to the traditional Linux model, shared libraries in Android are relocatable ELF files that map to the address space of the process when loaded. To save memory and avoid code duplication, all shared objects shipped with Android are dynamically linked against the Bionic libc library [23]. The shared libraries will be loaded into arbitrary memory space. To resolve the address of unknown symbols at load time, the dynamic linking technique is used.

The main parts of ELF format are the ELF file header, section header table (SHT), and program header table (PHT). At the beginning of the ELF file, there is a fixed-length file header which includes the starting position and length of SHT and PHT in the current ELF file. ELF organizes and manages information in sections. ELF uses SHT to record basic information for all sections. It mainly includes the type of section, the offset in the file, the size, the relative address of virtual memory after loading into memory, the alignment of bytes in memory and so on.

For an ELF file that is dynamically linked to other shared object files, to call the shared object functions, it is an instruction to jump to some stub function in the PLT table. In order to retrieve the real address of the target function, the stub function then performs a memory load on the entry in the GOT table. That is, the GOT table contains an array of all dynamically linked external functions’ pointers that are referenced by their code. In the period of dynamic linking, the GOT table is filled with the right function pointers depending on the maps between GOT entry and the function in shared object files, in the control of the metadata stored in the ELF file.

The PLT hook exploits this level of indirection introduced by dynamic linking. It goes through every loaded ELF file and overwrites its GOT entries with the pointer to a user-defined method, which is equivalent to performing dynamic linking another time but substituting the method’s pointer.

### 2.2. Dynamic Dispatch in C++

In this section, this paper briefly reviews how dynamic dispatch invokes object methods in C++. C++ dynamic dispatch rules dictate that when a virtual method is called, the real implementation depends on the runtime type of the calling object. This layer of indirection allows subclasses to override their parent class’s implementation of methods and is one of the key mechanisms for polymorphism in C++. Whether the parent’s method or child’s method will be invoked depends on what the object refers to at runtime.

Virtual Method Tables (vtable) are used commonly in dynamic dispatch implementation strategies. Because of their efficiency, popular C++ compilers, such as GCC, Visual C++, and Clang++, all use vtables. Vtable is a table that stores the addresses of the virtual methods in a class. To implement vtable, the compiler assigns an identifier to each virtual method of a C++ class, we assume it is the method’s serial number. The vtable is an array whose item represents the corresponding method of the class. When compiling the code, a vtable is constructed by the compiler for each class, and initialize the first word of the constructed object with a pointer to the vtable by inserting code in the class’s constructor.

**Figure 1:**
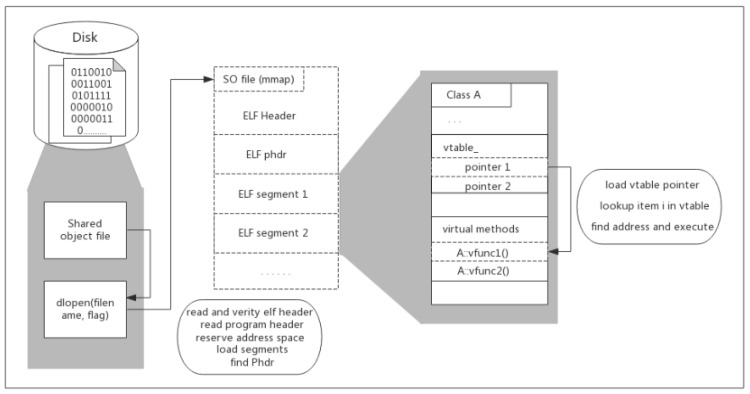
Shared object loading and inner method execution.

To implement call for a virtual method, the compiler has to perform these steps: first, load the vtable pointer which is located at the initial position in the calling object. Then, look up the item with index i in the vtable, where i is the serial number of the called method. Last, invoke the method implementation whose address is stored in the vtable’s item. Figure 1 shows how the shared libraries are loaded and the inner virtual methods are executed in the runtime.

## 3. Framework Design

The goal of this framework is to develop a generic native hooking method that is valid for different apps subject to security controls without modifying both the Android system and app’s code. Therefore, the design of this method is oriented towards modifying the target native methods’ runtime address in memory after the corresponding SO file is loaded into the app’s memory space and the relocation period. This framework contains two components. The first component is the core hooking engine and the user-defined code (also called patch code) written in C which will be compiled to a SO library. The other one is the Java side that is used for loading the former SO library and performing the hijack operation. The core engine aims to modify the content in reference address which points to the target method to the address of patch code. Moreover, it stores the original native methods’ entry address in ELF. The user-defined methods should have the same parameters form with the target ones.

Suppose that you desire to intercept calls to a native method in a shared library. For example, it contains vulnerable code, or it can actually deal with sensitive data. You have to define your own C method and override the target method by using the native hook method described in this paper. All calls to the target method will be intercepted and then go to your C method. This hooking framework supports loading and running patch code from the shared library. Because the hook core engine and the patch code both written in C, the hook program’s execution is more efficient.

This hooking framework is based on the virtual environment and uses the VirtualXposed (VXP) tool [25]. Normally, if one desires to hook other processes, the root privilege is needed to modify the app’s virtual memory or inject the hooking library in the running app or the zygote [26] master process [27]. The VXP tool is used to load and run the SO library which works as the core hooking engine and contains the patch code. Both the target process and the hook code are running in the same virtual environment. Therefore, the hooking library is able to modify the target process’s address space without the root privilege.

**Figure 2:**
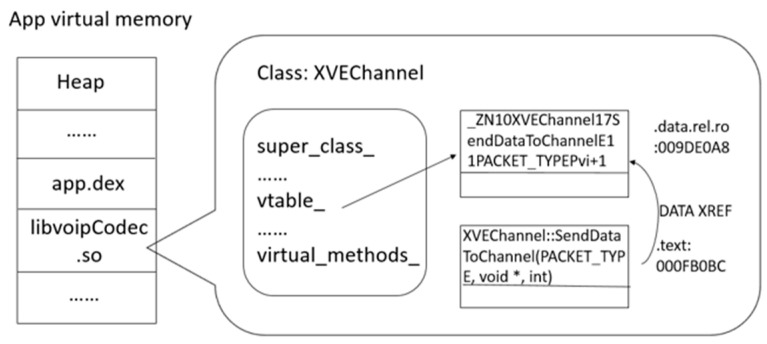
The application’s memory layout without hooking a method.

Now, we explain this native hook method design in figures by choosing the WeChat app as an example. Figure 2 illustrates the app’s memory layout without hooking. The libvoipCodec.so file which is a shared library of the app has been loaded into memory. The external and internal native methods are referenced in the addresses where they contain the functions’ pointers. *SendDataToChannel* method is an internal virtual method, therefore its reference is in the vtable in this example.

**Figure 3:**
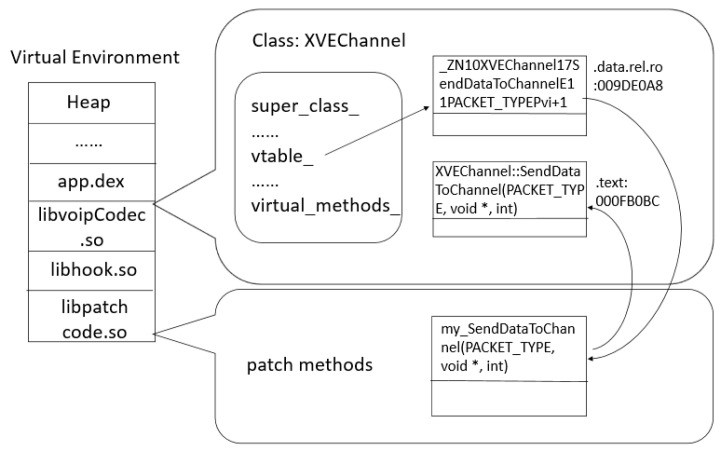
The application’s memory layout with hooking a method.

Instead, Figure 3 represents the app’s memory layout while the hooking library is enabled. First, the hooking libraries are loaded inside the virtual environment which also contains the target app. After this, the hooking method uses its internal functions to calculate the absolute address of the reference items which point to the target methods. Thereby it can hijack the methods by address substitution.

## 4. Implementation

Our hooking approach’s prototype implements each of the components described in Section 3. In the following, we describe the implementation details as well as a case study using this method.

### 4.1. Patch Native Method

The main responsibilities of this method’s core engine are the reference address calculation and rewriting in order to replace the vulnerable method with the patch code. Once the target program is loaded into memory, the core engine collects and inspects the properties of the shared objects via the “/proc/<pid>/maps” file, which is used to map dynamically linked libraries used by the executable file. The main SO file’s properties collected include the filename and the base address of the ELF in memory. After the SO information is collected, the resolute address of the target method’s reference in memory can be calculated by adding the ELF’s base address and the offset of the reference in ELF.

After the absolute address is obtained, this approach performs the inline hooking operation by overwriting the target native method’s entry address in the reference item, such as .data.rel.ro section and .got section in ELF, to the address of the method’s patch code.

To guarantee reliable hooking, this method first verifies the offset address of the target method defined in the ELF’s .text section with the address stored in the relocation item which has been modified to absolute address after the relocation period. After the binary and operation is executed between those addresses and 0xffe, the result should be the same and then the target absolute address can be modified.

To access the memory space, the read and write permissions are required by calling the mprotect method which is in units of pages. After the entry address is rewritten, since the processor might cache the instruction, to make sure the hijack operation takes effect, the __builtin___clear_cache method is performed to clear the processor’s instruction cache which is also in pages and has the processor read those instructions from memory again.

The original method is not modified by this framework and its address is stored inside this framework’s program, which will be used to call the original method implementation and get the return value of it. When this framework hooks a target method, all calls to that method will be intercepted and they will go to the patch code. Then, the patch code receives the target method’s arguments as its own parameters. Internally, this framework can invoke the original implementation of the target method by the method’s address stored before the hooking phase.

The core engine and patch code will be compiled to shared libraries in different application binary interface (ABI) depending on the phone’s Central Processing Unit (CPU) type. The current mainstream Android devices are based on armeabi-v7a architecture.

### 4.2. Combine this Method with VXP

In terms of deployment of the security policies using this inline hooking method on non-root devices, this study combines this method with VXP to achieve hijacking the other application’s process. This section needs to load and launch the hooking shared libraries, which is the core engine of this framework and the patch code in the VXP’s process at its startup and performs the hooking operation continuously in the background.

Once the hooking shared libraries have been loaded, the hooking points registration is implemented via the “start” function. The hooking method information includes the SO’s filename that the method belongs to, the offset address of the original method’s implementation, the offset address of the reference in ELF, and the user-defined patch method.

After the registered information is collected, in order to extract the shared libraries’ information of the target process which is running in the VXP’s virtual environment from memory, the native method “refresh” is called to read the maps information. Depending on the target method’s information registered before, it determines which patches should be deployed and then performs the hook operation as introduced in Section 4.1. In particular, the method “refresh” is invoked every several seconds in the background to hook the applications that are installed and launched on VXP later.

### 4.3. Case Study

Now, we demonstrate a case study by achieving WeChat call’s encryption functionality with the Advanced Encryption Standard (AES) algorithm in this framework. This feature is mainly implemented by the following steps: (a) analyzing the shared libraries and locate the target methods’ addresses; (b) providing the patch methods and other hooking information in this framework and compiling it into SO libraries; (c) loading and launching these hooking libraries from VXP. We describe these steps in detail in the following.

To implement the call’s encryption, the target method *SendDataToChannel* is chosen and located in the libvoipCodec.so file which is responsible for the digital signal processing by means of the IDA tool. The tag of “DATA XREF” in IDA is useful for obtaining the reference position of the method. The confirmation information is requested by this method which contains the definition of methods to hook, which are introduced in Section 4.1. The patch code is called instead of method *SendDataToChannel*, some parameters of which represent the pointer to the call data’s buffer and the length of the data, respectively.

Therefore, the encryption process in AES Counter Mode (AES-CTR) can be performed by utilizing these parameters. A unique per-packet value required by AES-CTR is called an Initialization Vector (IV) and it is generated by each Real-time Transport Protocol (RTP) packet header in this study. Therefore, the combination of the IV and key is not same in any encryption process. The AES block cipher is used by AES-CTR to create a stream cipher and the sensitive data is encrypted and decrypted by XORing with the key stream produced by AES encrypting sequential counter block values. Using streaming cipher instead of block cipher does not increase the length of RTP packets.

To restore the original call-flow with the modified parameters, the original method’s address stored in the core engine is available. Moreover, the decryption process should be implemented on the receiving end by hooking the method of receiving RTP packages accordingly. After loading and running this framework in VXP, all future calls to method *SendDataToChannel* will be redirected to the patch code.

## 5. Evaluation

In this section, we present an evaluation of this native hooking framework. The goal of the evaluation is threefold: (a) to demonstrate the functionality which needs to be tested whether this has extensive use, (b) to measure the performance overhead incurred by this method by comparing the overhead between the hook and unhook, and (c) to demonstrate the compatibility with existing applications and different Android versions of mobile phones.

### 5.1. Functional Evaluation

Regarding the functional evaluation, we conducted experiments on different apps which are taken from the official market to enhance their security. Specifically, voice call encryption function is achieved on three popular social apps: WeChat, Line, and WhatsApp, using this method to prevent call data leakage during transmission. Our methodology for this evaluation was to: (a) analyze the shared object related to VOIP of the target apps by means of the IDA tool and obtain the appropriate methods as hook points; (b) install this framework as well as the patch methods; (c) and, run the call function to determine whether the encryption is performed successfully. Similarly, another experiment is based on the Canon Camera Connect application which can transmit the pictures from the camera to the phone. The picture encryption is performed to prevent sensitive data from being written directly into the phone’s storage space by hooking the file manipulation methods. Table 1 manifests the target methods of these target apps.

As shown in Table 1, these target methods in four apps all can be hooked and patched successfully using the method proposed in this paper to achieve the data protection purpose. A multitude of underlying methods that deal with sensitive data of Android applications is defined or used in shared libraries. These native methods may be exploited by hackers to disclosure users’ privacy. The conclusion can be drawn from the experiments that this native hooking method is able to enforce security and privacy policies and has wide usage.

### 5.2. Performance Overhead

**Figure 4:**
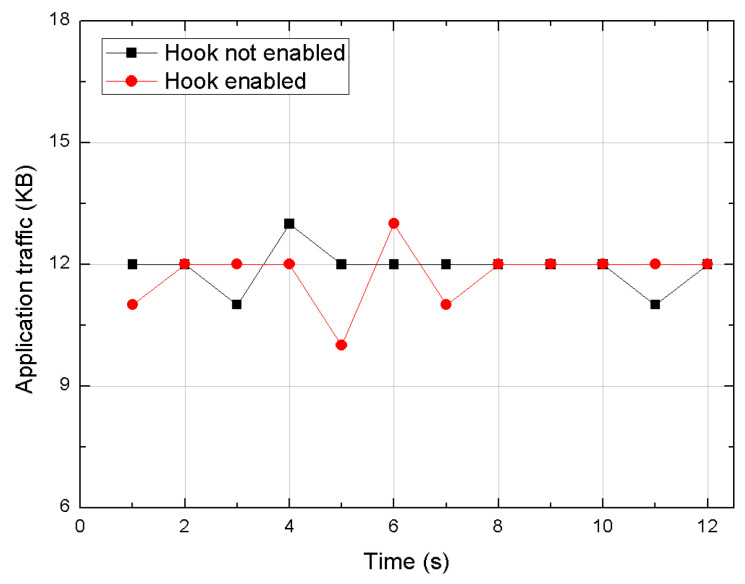
Traffic comparison on WeChat application.

**Figure 5:**
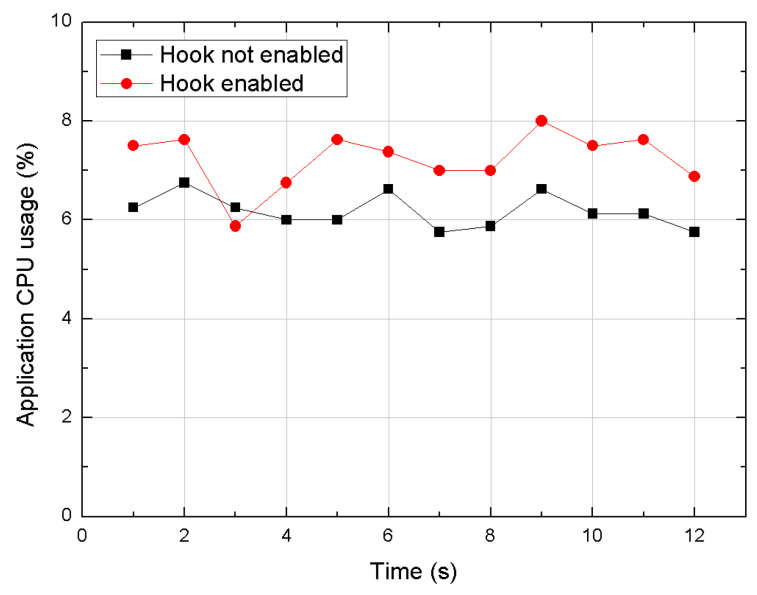
CPU usage comparison on WeChat application.

The goal of our performance evaluation was to determine if significant overhead is imposed on a real-world application by running this hooking method on an Android device. This study compares the network traffic and the CPU usage on WeChat application before and after hooking the packet sending and receiving methods during voice call using this framework. As shown in Figure 4, the network traffic turns out to be almost unaffected by the hooking operation. Regarding the CPU usage, Figure 5 shows that there is only a minimal and negligible overhead added through this method.

Native methods are often used to deal with underlying data and achieve high-performance application logic, which may be executed frequently. To check the impact of intercepting the methods of sending and receiving packets using this framework on voice call function, we measure the number of the packets sent and received per second on WeChat, Line, and WhatsApp apps shown in Table 2, once with the hooking method disabled on one part and count on the other part, and then with the hooking method enabled both sides. These small decreases in packet number are not noticeable by the user in any way.

**Figure 6:**
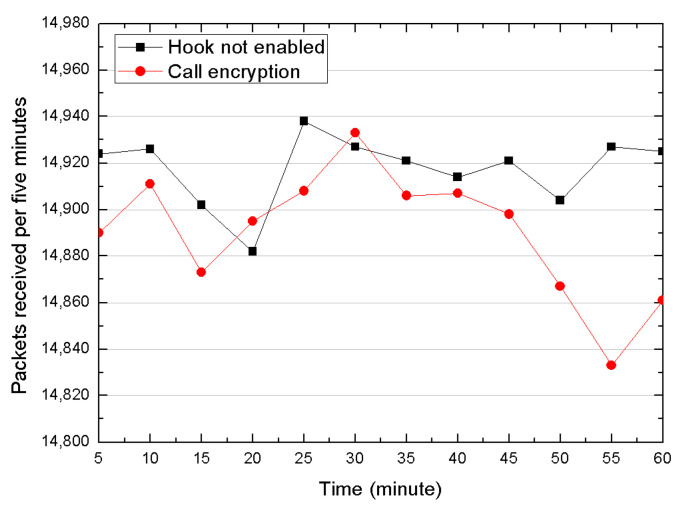
Packet traffic comparison per five minutes.

The overhead introduced by our method depends much on the behavior of the patch code. Take the WeChat call encryption function as an example, we further compare the number of packets received every five minutes in a similar way but enforce AES256 encryption policies on both sides when hook is enabled. The result is shown in the following Figure 6. The result indicates that the most of the overhead in this method is triggered by encryption and decryption operations. In this case, the average packets number is 14,918 with unhook and 14,890 with call encryption, that is, there are 28 packages that are delayed or lost. The rate of delaying or losing is 0.19%, which we believe is acceptable. In objective testing, we did not feel any lagging when the call encryption was performed. The above experiments fully demonstrate that this hooking approach has a relatively small impact on the application and the system.

### 5.3. Compatibility Evaluation

The previous section conducted experiments on the Samsung G973F Android 9.0. To gain greater assurance in this method’s compatibility, we installed and ran this framework on different Android devices. Table 3 shows the devices for our evaluation. The HUAWEI Mate10 Pro is equipped with different processors from Samsung. In addition, the three Samsung phones run different major Android versions (8.0, 9.0, 10.0). The result of this study shows that this framework is able to hook security-sensitive native methods on these devices without any problem.

The core of this native hooking method is to calculate the reference address of the target method at runtime and rewrite it. As long as the virtual memory which contains the target ELF can be read and written, this hooking method can be used. Furthermore, the VXP supports the Android version from 5.0 to 10.0. Therefore, one can use this app to run our hooking method without root permission on these different Android versions, and this hooking method is compatible.

## 6. Discussion

We note that the main goal of our work is to propose a novel technique to hook the native methods in shared libraries, our approach can be used to enforce fine-grained security policies to prevent sensitive information leakage during transmission on real-world non-root devices. In contrast to injecting shared libraries into the target process, our approach uses the VXP tool which is based on the sandboxing mechanism and allows the target app as well as the patch libraries running in the same virtual environment without requiring root privileges.

In Section 5, we presented an evaluation about the functionality of this approach by patching security issues on social apps. Since the ELF structure of the diverse SO libraries is consistent, this approach is generic and has wide usage. Furthermore, the VXP framework can run a variety of Android applications, consequently this framework can be applied to a multitude of apps.

Obviously, this native hooking method has its limitations and corner cases. The main limitation is due to the choice of hooking points. There are a variety of internal functions and export functions defined in the shared objects. The proposed method is suitable for hooking all internal and export functions with function pointers in different applications. In a scenario where the target method for processing sensitive information does not have the function pointer, it might not be intercepted successfully. In addition, developers need to manually locate the target functions’ addresses, entry parameters, and so on by means of analyzing the shared objects. Moreover, regarding the VXP tool, it possibly has compatibility issues on different Android devices.

## 7. Related Works

Several approaches have been proposed to provide methods hooking the native methods on Android. Frida [28], a dynamic instrumentation toolkit and Cydia substrate [24] are able to replace methods in native binaries as well as the Dalvik bytecode. These tools are based on device modding and the system components, such as zygote, require to be replaced. In fact, both Frida and Cydia substrate are not able to work on non-root devices. Furthermore, a server program is needed on Frida to install on phone to communicate with the script on the PC. Therefore, it is not suitable for practical production.

Aurasium [23] builds a reference monitor into application binaries and rewrites function pointers in a module’s global offset table. Clearly, such approaches are not effective if the vulnerable methods are internal in shared libraries. In addition, when the application is patched statically and repacked, the package signature is broken. By contrast, our approach does not need the APK repackaging but only modifies in the memory space, thus we do not break the code signature. Mulliner et al. proposed PatchDroid [29], which is a system to distribute and apply third-party patches for security vulnerabilities in native and managed code in Android. Its patch method for native code is based on shared library injection and therefore the root privileges are required to be able to attach to processes such as zygote.

Several approaches aim to achieve hooking on ART Runtime. You et al. proposed TaintMan [30], an ART-compatible DTA framework that can be deployed on unmodified and non-root Android devices by instrumenting taint enforcement code into the target application and the system class libraries to track data flow and control flow. ARTDroid [31] proposed a virtual method hooking framework on Android ART Runtime by tampering vtable to monitor sensitive methods. ArtHook [32] proposed a dynamic callee-side rewriting approach by changing the prologue of the method being hooked, and redirect the call to another method leaving most of the original method intact. Epic [33] is also based on rewriting the method’s entry point by changing the first 8 bytes in memory to a jump instruction. TaintART [34] proposed a multilevel information-flow tracking system. While these approaches are valuable, ART hook is more at the virtual machine level in Android and used to intercept Java methods.

App sandboxing is an important means of sandboxing unmodified apps in non-rooted devices on stock Android. Michael et al. proposed Boxify [22], an approach based on application virtualization and process-based privilege separation to encapsulate unmodified apps in an isolated execution environment within the context of another. Bianchi et al. proposed NJAS [35], similar to Boxify, an approach to sandbox arbitrary Android applications by means of system call interposition.

## 8. Conclusions and Future Work

Security-sensitive data leakage can occur through many Android applications. In this paper, we present a practical framework for hooking native methods in shared libraries in Android. This framework supports the method hooking without any modifications to both the Android system and app’s code by reference modification and control flows redirection and is thus easy to deploy. The final form of this method is shared object files that contain the core engine and the patch methods. We combine this method with VXP and install the target App inside the VXP’s virtual environment, and the hooking operation can be performed without root privilege on any real devices. This framework can be used to analyze Android malware, patch the security vulnerabilities, and implement security policies. Evaluation result indicates that there is a considerable amount of sensitive methods that can be hooked, and therefore this framework can effectively enhance application security. In addition, it does not produce any significant runtime performance overhead on real devices and it is compatible with different versions of Android devices and applications, thus it is suitable to be used in the real world. Our current approach is restricted to hook the native methods within the user applications. As future work, we plan to extend our methods to sensitive native methods in the system framework.

## Figures and Tables

**Table 1:** Target methods of four apps.

Application	Hook Method	Reference Section	Whether Can Be Hooked
WeChat	SendDataToChannel	.data.rel.ro	√
	XVE_ReceiveRTPPacket	.data.rel.ro	√
Line	Srtp_cipher_encrypt	.got	√
	Srtp_cipher_decrypt	.got	√
WhatsApp	transport_send_rtp2	.data	√
	srtp_rtp_cb4	.text	√
Canon Camera Connect	fopen	.got	√
	fwrite	.got	√

**Table 2:** Performance on three social apps.

Application	Received Packets without Hook	Packets Sent without Hook	Received Packets with Hook	Packets Sent with Hook
WeChat	49.969	50	49.787	50
Line	3.667	4.009	3.55	3.943
WhatsApp	15.967	15.589	15.529	15.34

**Table 3:** Experimental results of Samsung and Huawei in different Android versions.

Phone	Samsung G960	HUAWEI Mate10pro	Samsung G973F	Samsung G973F
Operating System	Android OS 8.0	Android OS 8.0	Android OS 9.0	Android OS 10.0
CPU	Exynos 9810	Kirin 970 2417MHz	Exynos 9820	Exynos 9820
CPU Architecture	Armeabiv-v7a	Armeabiv-v7a	Armeabiv-v7a	Armeabiv-v7a
Whether Root	No	No	No	No
Compatible with VXP	Yes	Yes	Yes	Yes
Native Hook Result	Success	Success	Success	Success
